# Supplementary material for: SPUMONI 2: improved classification using a pangenome index of minimizer digests
Source: Genome Biol. 2023 May 18;24:122. doi: 10.1186/s13059-023-02958-1 (PMC10197461; doi:10.1186/s13059-023-02958-1)
Supplement: Supplementary file 1 — Additional file 1. SPUMONI 2 Supplement. This document contains supplementary tables and figures. [file 13059_2023_2958_MOESM1_ESM.pdf]

SPUMONI 2 Supplement

Omar Ahmed[1], Massimiliano Rossi[2], Travis Gagie[3], Christina Boucher[2], and Ben Langmead[1,*]

[1]Department of Computer Science, Johns Hopkins University
[2]Department of Computer Science, University of Florida
[3]Faculty of Computer Science, Dalhousie University
[*]*corresponding author;* `langmea@cs.jhu.edu`

April 28, 2023

Supplementary Figures

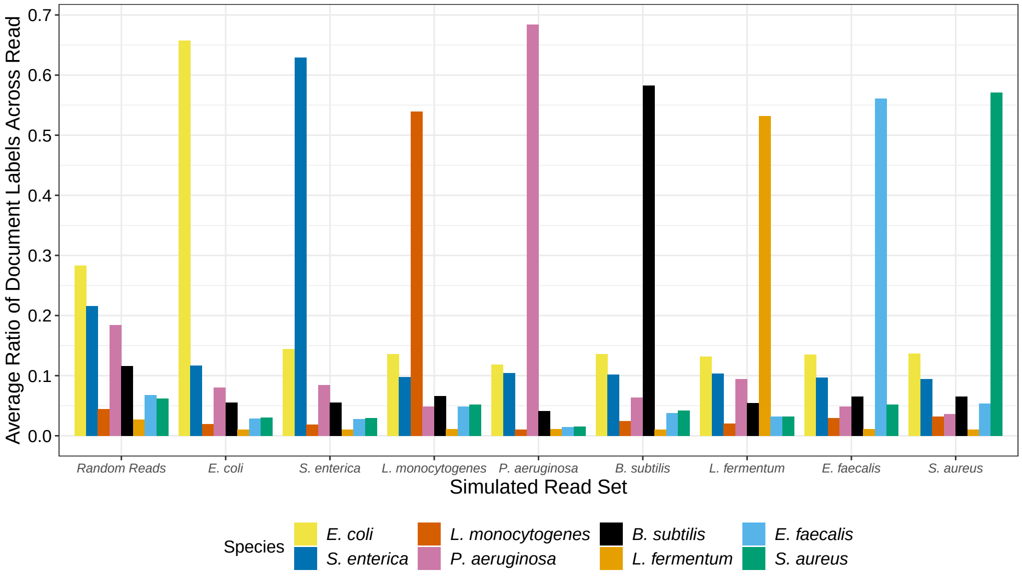

Figure S1: Shows the average ratio of document labels found at the read level when querying simulated long reads (ONT) from eight different microbial species against a pan-genome database of microbial species.

Algorithm 1 Computing pseudomatching lengths and document labels

```
given text T and a pattern p
j ← 1, ℓ ← 0, doc ← 0
for i ← p.len to 1 do
    if p[i] ≠ BWT[j] then                          ▷ Case 2 - Grab new document id
        if pos < Thr(j, p[i]) then
            new_j ← BWT.pred(j, p[i])
            doc ← DocArray.pred(j, p[i])
        else
            new_j ← BWT.succ(j, p[i])
            doc ← DocArray.succ(j, p[i])
        end if
        ℓ ← 0
    else
        ℓ ← ℓ + 1        ▷ Case 1 - Keep the same document id as previous step
    end if
    j ← new_j
    j ← LF(j)
    PML[i] ← ℓ, DocID[i] ← doc
end for
Return PML, DocID
```

Figure S2: Shows the pseudocode for computing pseudomatching lengths as well as document labels for a pattern P with respect to a text T.

Supplementary Tables

"Contaminant" Database Contents	
Species:	Number of Genomes:
Escherichia Coli	500
Saccharomyces cerevisiae	1

Table S1: Shows the the different species and the number of genomes used in the "contaminant" database for the assembly contamination experiment. All of the strains for the species listed above were obtained from the NCBI RefSeq database.

Pan-genomic database for small multi-class classification experiment		
Species:	Number of Genomes:	Number of Bases:
Escherichia Coli	2495	12.9 billion
Salmonella enterica	1309	6.40 billion
Listeria monocytogenes	281	833 million
Pseudomonas aeruginosa	470	3.15 billion
Bacillus subtilis	217	899 million
Lactobacillus fermentum	40	85.2 million
Enterococcus faecalis	459	1.38 billion
Staphylococcus aureus	861	2.44 billion

Table S2: Shows the eight microbial species used in the document array experiment, and the number of genomes from RefSeq that were included in the database. All of the strains for the species listed above were obtained from the NCBI RefSeq database.

Query times for contigs w.r.t to "contaminant" database	
Approach:	Time (speed-up):
SPUMONI 1	719.53 s (1.59)
SPUMONI 2	266.64 s (4.29)
Blast+	1144.55 s (1.00)

Table S3: Shows the time taken to query the assembly with respect to "contaminant" database for each method in seconds. Each tool was run with 16 threads. All the tools agreed in terms of which contigs had suspicious similarity to the "contaminant" database.

Example of SPUMONI 2 document array for an input text				
i	BWT	DA	SA	$T[SA[i]]$
0	A	2	45	$
1	G	2	44	A$
2	T	0	14	AAAATGGCCTACTAGAATTTTTAAATGTAGA$
3	A	1	15	AAATGGCCTACTAGAATTTTTAAATGTAGA$
4	T	2	36	AAATGTAGA$
5	A	1	16	AATGGCCTACTAGAATTTTTAAATGTAGA$
6	A	2	37	AATGTAGA$
7	G	1	29	AATTTTTAAATGTAGA$
8	T	0	10	ACATAAAATGGCCTACTAGAATTTTTAAATGTAGA$
9	T	-	-	ACTAGAATTTTTAAATGTAGA$
10	T	-	-	ACTAGATACATAAAATGGCCTACTAGAATTTTTAAATGTAGA$
11	T	-	-	AGA$
12	T	-	-	AGAATTTTTAAATGTAGA$
13	T	0	6	AGATACATAAAATGGCCTACTAGAATTTTTAAATGTAGA$
14	C	0	12	ATAAAATGGCCTACTAGAATTTTTAAATGTAGA$
15	G	0	8	ATACATAAAATGGCCTACTAGAATTTTTAAATGTAGA$
16	A	1	17	ATGGCCTACTAGAATTTTTAAATGTAGA$
17	A	2	38	ATGTAGA$
18	$	0	0	ATTACTAGATACATAAAATGGCCTACTAGAATTTTTAAATGTAGA$
19	A	2	30	ATTTTTAAATGTAGA$
20	A	0	11	CATAAAATGGCCTACTAGAATTTTTAAATGTAGA$
21	G	1	21	CCTACTAGAATTTTTAAATGTAGA$
22	C	1	22	CTACTAGAATTTTTAAATGTAGA$
23	A	1	25	CTAGAATTTTTAAATGTAGA$
24	A	-	-	CTAGATACATAAAATGGCCTACTAGAATTTTTAAATGTAGA$
25	A	-	-	GA$
26	A	-	-	GAATTTTTAAATGTAGA$
27	A	0	7	GATACATAAAATGGCCTACTAGAATTTTTAAATGTAGA$
28	G	1	20	GCCTACTAGAATTTTTAAATGTAGA$
29	T	1	19	GGCCTACTAGAATTTTTAAATGTAGA$
30	T	2	40	GTAGA$
31	A	0	13	TAAAATGGCCTACTAGAATTTTTAAATGTAGA$
32	T	2	35	TAAATGTAGA$
33	A	0	9	TACATAAAATGGCCTACTAGAATTTTTAAATGTAGA$
34	C	1	23	TACTAGAATTTTTAAATGTAGA$
35	T	0	2	TACTAGATACATAAAATGGCCTACTAGAATTTTTAAATGTAGA$
36	G	2	41	TAGA$
37	C	1	26	TAGAATTTTTAAATGTAGA$
38	C	0	5	TAGATACATAAAATGGCCTACTAGAATTTTTAAATGTAGA$
39	A	1	18	TGGCCTACTAGAATTTTTAAATGTAGA$
40	A	2	39	TGTAGA$
41	T	2	34	TTAAATGTAGA$
42	A	0	1	TTACTAGATACATAAAATGGCCTACTAGAATTTTTAAATGTAGA$
43	T	2	33	TTTAAATGTAGA$
44	T	2	32	TTTTAAATGTAGA$
45	A	2	31	TTTTTAAATGTAGA$

Table S4: Shows the SPUMONI 2 document array for the collection of documents $T = \{\text{ATTACTAGATACATA, AAATGGCCTACTAGA, ATTTTTAAATGTAGA}\}$. The document array stores the class number that the suffix at the run boundaries occurs in. The gray rows in the table are labeling the run boundaries of the BWT, which are the ones containing document-array entries. Note that the document array will be much sparser for a larger, more repetitive text.

Supplementary Notes

Read Simulation

In the paper, read simulators such as Mason and PBSIM2 were used to simulate short and long reads respectively. The commands used to generate those reads are shown below:

```
mason_simulator -ir $genome -n $num_of_reads -v \
                -o $output_read --illumina-read-length 150 \
                --illumina-prob-mismatch 0.01
```

This command above was generally used throughout the paper for simulating short reads with 1% mismatch error. Next, the command below shows how long reads were simulated with 95% read accuracy using R9.4 chemistry.

```
pbsim --depth 50.0 --prefix $output_prefix \
      --hmm_model R9.4.model  --accuracy-mean 0.95 \
      $positive_genome
```
