# Supplementary material for: SPUMONI 2: improved classification using a pangenome index of minimizer digests
Source: Genome Biol. 2023 May 18;24:122. doi: 10.1186/s13059-023-02958-1 (PMC10197461; doi:10.1186/s13059-023-02958-1)
Supplement: Supplementary file 2 — Additional file 2. Review history. [file 13059_2023_2958_MOESM2_ESM.docx]

**1^st^ round**

**Reviewer 1**

Review of "SPUMONI 2: Improved pangenome classification using a compressed index of minimizer digests" by Ahmed et al

In this paper, Ahmed and colleagues set out to develop new algorithms/data structures for read classification in comparison
with a large database/set of reference genomes, building on their prior work (Spumoni and MONI).

The problem they wish to solve is of wide interest - read classification is heavily used in the context of taxonomic assignment
(eg to estimate species abundances in metagenomes, or to separate host DNA from microbial DNA). The authors are also interested
in applying this to human genomes, where the intent (as I understand it) is to find the best matching human reference
to the read. Previously, the authors developed a tool called Spumoni, which performed binary classification of reads
by comparing with an r-index (augmented specialised BWT) and collecting so-called match statistics, which were cheaper
to calculate than aligning, but informative enough to detet approximate matches. This paper sets out to improve spumoni
- reduce RAM use, allow multi-class classication, scale better, handle illumina data also. Their key innovations
(all of which are clever and will doubtless be studied for some time to come) are
1. to convert each reference genome to a list of minimizers, concatenate them, and then create the r-index of this.
2. using approximate match statistics
3. I think the idea of storing class (taxonomic) information throughout the BWT at run boundaries is super-interesting.

The authors succeed in their goal; the paper is succinct, mostly clear and well benchmarked - I congratulate them!
I have a number of issues that need clarification, but it is clear this is an valuable contribution to the field.
I must apologise that I have not tried to install and run the software - I figured getting the review in before xmas was a bigger win than a delay for me to do that.


Major comments

1. I am a little hazy on the sampled document array, for a few reasons.

a) For bacteria, i can see that the class is going to be taxonomic info, probably species (right? seems so from the final figure).
I'm not sure what the class is for the human pangenome example, is it just the identifier of the human genome?

b) What does this mean exactly: "SPUMONI 2's sampled document array consists of 2r integers encoding the class of the
suffixes at the beginning and end of every BWT run."
- you need 2r because there are r runs and you store an int at both ends? Even though the end of one run is the start of another?
Is this basically because many runs are just one character long?
- what does "the suffixes at the end of a run" mean actually? In Supp Table 4, for example there is a run (of one character) at i=3.
Is the suffix at the end of that run T[SA[3]] ? Maybe a clearer example is the polyA run between i=23 and i=27. At i=23, you store
1 in the DA column, which means the second reference as you seem to be zero indexed. Are you storing
the class of CTAGAATTTTTAAATGTAGA$ ? But that string is not in any of the references? Ah - reverse complement? You append
the reverse complements before making the BWT and give them the same class labels as the original?


2. Section 4.4 - since you have only 256 minimizers, you can use a minimal perfecr hash anyway right? no need for Rolling Hasher?
I guess also no big cost, and you have a reliable library and it will work if you change k.


3. Table 1 is showing statistics for indexing 500 E coli genomes. That ought to be a reference of length = 500 X 5Mb = 2.5 billion bases.
But you show, for n in the No digestion column, 5 billion? Are you indexing both the genomes and their reverse complements?


4. Caption of figure 5 should explicitly say this is for illumina reads.

5. Why do you think your match class ratio histogram is less informative (the true taxon still wins, but by less) for long nanopore reads?

"we find that between 80.7% and 88.0% of the classes reported by the sampled document array at the read level match the true
class of origin. For long ONT reads, we see a similar pattern where the true class is in the majority, ranging from 53.8% to 66.3%"

My only guess is long reads are more likely to have mobile elements that are shared across species, but i'd be surprised it made that much difference.
How long were your nanopore reads?


6. Figure 4 - can you use histograms or something instead of boxplots? V unclear how many data points there are. Can you explain why you say having an average PML>=2
makes them suspicious?


Arguable major/minor comments
- Personally, i would suggest you change the title of the paper slightly. The current title seems to be saying spumoni2 is a tool for
classifying pangenomes ("Improved pangenome classification").


Minor comments


1. K-mer size. When i saw in Figure 1 that you were using k=4, i thought k=4 was purely illustrative, but realised with amazement later on that it was not. But i think this is tremendously interesting - instead of choosing such a large k that the k-mer is intended to be taxonomically informative (as many read classifiers do), you simply
choose a light-weight encodable minimizer scheme that allows you to sparsify.

BTW i think this sentence in the Figure 1 caption is wrong, or ambiguous

"The k-mer that minimizes a hash function in each w-bp window as well as being distinct from the previous minimizer is chosen and represented as an 8-bit character in the new reference."

I don't think you take a w-window, and find the k-mer which minimizes the hash function, and if it is not distinct from the previous minimizer, get another kmer from the same window.
I think you get one minimizer per window and "homopolymer compress". That text looks like you choose something from each window which minimizers and is not the same as the last one.
Sorry, v fussy detail.


2. Page 4, section on Minimizer digestion - maybe good to explicitly say that with k=4, there are 4^4 = 256 possible k-mers and therefore minimizers, so you can encode that in 8 bits.

**Reviewer 2**

The manuscript "SPUMONI2" by Ahmed et al presents a significant improvement over the previously published tool, SPUMONI for read classification problems including taxonomic classification, host sequence depletion, adaptive sampling of long nanopore reads, and accurate detection of contaminants in reference databases.

The authors have developed a multi-class read classifier that is both faster and more memory-efficient. By utilizing a novel sampled document array structure, the tool is capable of efficiently solving read classification problems for both short and long reads. The results show a three-fold increase in speed and a two-fold reduction in index size compared to SPUMONI. The authors also demonstrate the tool's ability to effectively identify contaminated contigs in genome assemblies, further highlighting its versatility and usefulness.

The authors have effectively validated the performance of SPUMONI2 using different datasets such as 500 Escherichia coli genomes, a mock community dataset, and the Human microbiome dataset, and compared it with SPUMONI and minimap2. They found that SPUMONI2 has a 15x faster classification speed for both long and short reads in the mock community dataset with a far smaller index size compared to minimap2.

However, it should be noted that the improved speed and memory footprint come with a slight reduction in classification accuracy. To better evaluate the impact of minimizer digestion on classification accuracy, one suggestion would be to include additional data points for classification accuracy using additional window sizes 10, 11, and 12. Furthermore, a deeper analysis of the regions that the tool struggles to classify correctly using SPUMONI2's minimizer digestion could provide valuable insights into the complexity of these regions.

Overall, the SPUMONI2 tool is a valuable addition to the read classification problem and the authors should be recognized for their efforts.

Minor Comments:
Move Table 2 after section 2.4.
In section 2.5, line 36, delete one "using" from a sentence.
Move Figure 4 after section 2.5

**Authors’ response**

Reviewer #1 Comments:
Comment:
In this paper, Ahmed and colleagues set out to develop new algorithms/data structures for read classification in comparison with a large database/set of reference genomes, building on their prior work (Spumoni and MONI).
The problem they wish to solve is of wide interest - read classification is heavily used in the context of taxonomic assignment (eg to estimate species abundances in metagenomes, or to separate host DNA from microbial DNA). The authors are also interested in applying this to human genomes, where the intent (as I understand it) is to find the best matching human reference to the read. Previously, the authors developed a tool called Spumoni, which performed binary classification of reads by comparing with an r-index (augmented specialised BWT) and collecting so-called match statistics, which were cheaper to calculate than aligning, but informative enough to detet approximate matches.
This paper sets out to improve spumoni - reduce RAM use, allow multi-class classication, scale better, handle illumina data also.
Their key innovations (all of which are clever and will doubtless be studied for some time to come) are

1. to convert each reference genome to a list of minimizers, concatenate them, and then create the r-index of this.
   2. using approximate match statistics
   3. I think the idea of storing class (taxonomic) information throughout the BWT at run boundaries is super-interesting.

The authors succeed in their goal; the paper is succinct, mostly clear and well benchmarked - I congratulate them! I have a number of issues that need clarification, but it is clear this is an valuable contribution to the field.
I must apologise that I have not tried to install and run the software - I figured getting the review in before xmas was a bigger win than a delay for me to do that.
Response:
We agree with reviewer #1’s key takeaways and appreciate the comments.

Comment:
I am a little hazy on the sampled document array, for a few reasons.
a) For bacteria, i can see that the class is going to be taxonomic info, probably species (right? seems so from the final figure). I'm not sure what the class is for the human pangenome example, is it just the identifier of the human genome?
b) What does this mean exactly: "SPUMONI 2's sampled document array consists of 2r integers encoding the class of the suffixes at the beginning and end of every BWT run."
- you need 2r because there are r runs and you store an int at both ends? Even though the end of one run is the start of another? Is this basically because many runs are just one character long?
- what does "the suffixes at the end of a run" mean actually? In Supp Table 4, for example there is a run (of one character) at i=3. Is the suffix at the end of that run T[SA[3]] ?
Maybe a clearer example is the polyA run between i=23 and i=27. At i=23, you store 1 in the DA column, which means the second reference as you seem to be zero indexed. Are you storing the class of CTAGAATTTTTAAATGTAGA$ ? But that string is not in any of the references? Ah - reverse complement? You append the reverse complements before making the BWT and give them the same class labels as the original?
Response:
a) Yes, for the index of the bacterial references of eight different species used in Section 2.6, we would store a number from 0 to 7 at each run boundary to indicate what species was present at that position in the BWT.

As for the human pangenome experiment shown in Table 2, we were strictly performing binary classification for adaptive sampling, and so did not use a sampled document array. In general, though, it is up to the user at index-building time how they would like to label the sequences. A human pangenome could receive labels that distinguish individuals, populations, super-populations, etc.
b) Yes, so it is 2r because we have r runs and store an integer at the beginning and end of each run. The reviewer is correct that the end of one run of characters is followed by the start of another run. We store an integer at both since our algorithm (modeled on MONI) will sometimes use a "repositioning" step to move to a nearby run head or tail. Whether we reposition to a head or tail depends on the "threshold" value as described in the MONI paper. We need the destination of the repositioning to have a document label regardless of whether it is a head or tail. Here is a short example of the positions of the BWT where we would store a document id:
BWT = AAACCCTTTAAATTTGGG
Underlined characters correspond to positions that would have an associated document sample.
With regards to Supp. Table 4, for i=3, the suffix T[SA[3]] is considered to be the beginning and end of that run since the run is only 1 character. So in our implementation, since we store the document ids of start of the runs and ends of the runs separately as two different integer vectors, we would store the value DA[3]=1 in both vectors.
Yes, the document ids are zero-indexed. In a 3-document scenario, we would use 0, 1, and 2 to differentiate them in the document array.
Yes, so in terms of the suffix from i=23 (CTAGAATTTTTAAATGTAGA$), we store 1 meaning the 2nd document because the suffix array position SA[23] = 25 corresponds to a position in the 2nd document. As we process the query, we are finding exact matches at the start of these suffixes so that is why we take the suffix array position and convert it to a document id.
Yes, we do concatenate the reverse complement of each document, and then give them the same document id as the forward strand as we build the index. That allows us at query time to process the read, and find matches to either strand.

Comment:
Section 4.4 - since you have only 256 minimizers, you can use a minimal perfecr hash anyway right? no need for Rolling Hasher? I guess also no big cost, and you have a reliable library and it will work if you change k.
Response:
Yes, the reviewer is right since we are using only 256 minimizers for 256 possible 4-mers, we could use a minimal perfect hash, or simply a look-up table. We do use the RollingHasher object from the Bonsai library, chiefly because it is flexible enough to support various k-mer lengths, as the reviewer alluded to.

Comment:
Table 1 is showing statistics for indexing 500 E coli genomes. That ought to be a reference of length = 500 X 5Mb = 2.5 billion bases. But you show, for n in the No digestion column, 5 billion? Are you indexing both the genomes and their reverse complements?
Response:
Yes, we are indexing both the genomes and their reverse complements. We have added a note about this in Table 1 caption.

Comment:
Caption of figure 5 should explicitly say this is for illumina reads.
Response:
Thanks for pointing that out, we have added that to the caption in Figure 5.

Comment:
Why do you think your match class ratio histogram is less informative (the true taxon still wins, but by less) for long nanopore reads?
"we find that between 80.7% and 88.0% of the classes reported by the sampled document array at the read level match the true class of origin. For long ONT reads, we see a similar pattern where the true class is in the majority, ranging from 53.8% to 66.3%"
My only guess is long reads are more likely to have mobile elements that are shared across species, but i'd be surprised it made that much difference. How long were your nanopore reads?
Response:

The nanopore reads were simulated using the PBSIM2 software and its read length model has a mean of 9,000 bp with a standard deviation of 7,000 bp. We expected this decrease in the percentage of the document labels from the true class simply because of sequencing errors. More sequencing errors leads to shorter exact matches, which in turn give us less specificity in our document matches. Our simulated long reads had 95% read accuracy compared to 99% accuracy for the short reads.

Comment:
Figure 4 - can you use histograms or something instead of boxplots? V unclear how many data points there are. Can you explain why you say having an average PML>=2 makes them suspicious?
Response:
We agree with the reviewer that Figure 4 needed revising for clarity. First we will answer the question regarding the PML threshold, then describe how we changed the text and figure to address this point.
In this experiment, we built an index over E. coli and Yeast genomes, since they represent common contaminants. We computed pseudomatching lengths (PMLs) for each contig of the human assembly with respect to the index. The algorithm will compute a distinct PML at each position, meaning we get around 3 billion for a typical human assembly. In this particular experiment, we also applied the minimizer digestion method. Combining these factors: the number of the data points in each box plot equals the length of each contig after digestion.
After computing PMLs, we noticed the distribution for certain contigs of an assembly were shifted much higher than others. We hypothesized that those contigs had contamination, which yielded these longer-than-expected PMLs. By looking at the data, we observed that a PML threshold of >= 2, as used in the paper, produced a clean separation between “contaminated” contigs and the “uncontaminated” contigs. We now draw that line more clearly in Figure 4.
Returning to the first comment, we agree that the figure needed improvement. We have changed Figure 4a to be a scatter-plot where we plot a single point for each contig, with contig length on the x-axis, and the 25th percentile of the PML distribution for the contig on the y-axis. This makes it easy to interpret and visually we can see the threshold of 2 being applied to clearly find the contaminated contigs. All of these are now indicated more clearly with color and labeling.

Comment:

Personally, i would suggest you change the title of the paper slightly. The current title seems to be saying spumoni2 is a tool for classifying pangenomes ("Improved pangenome classification").
Response:
Yes, that’s a good point. Our new proposed title is "SPUMONI 2: Improved classification using a pangenome index of minimizer digests."

Comment:
When i saw in Figure 1 that you were using k=4, i thought k=4 was purely illustrative, but realised with amazement later on that it was not. But i think this is tremendously interesting - instead of choosing such a large k that the k-mer is intended to be taxonomically informative (as many read classifiers do), you simply choose a light-weight encodable minimizer scheme that allows you to sparsify.
Response:
That is correct. Informally speaking, we do not need any particular minimizer to be informative on its own, since we ultimately make classification decisions based on longer exact matches involving many minimizers that match consecutively.

BTW i think this sentence in the Figure 1 caption is wrong, or ambiguous
"The k-mer that minimizes a hash function in each w-bp window as well as being distinct from the previous minimizer is chosen and represented as an 8-bit character in the new reference."
I don't think you take a w-window, and find the k-mer which minimizes the hash function, and if it is not distinct from the previous minimizer, get another kmer from the same window.
I think you get one minimizer per window and "homopolymer compress". That text looks like you choose something from each window which minimizers and is not the same as the last one.
Sorry, v fussy detail.
Response:
Thanks to the reviewer for pointing this out. We did indeed mean to say we choose the minimizer from each w-bp window and then take just one representative from each consecutive stretch of identical minimizers. We have adjusted the caption to help avoid that possible confusion.

Comment:
Page 4, section on Minimizer digestion - maybe good to explicitly say that with k=4, there are 4^4 = 256 possible k-mers and therefore minimizers, so you can encode that in 8 bits.
Response:
Thanks to the reviewer for the suggestion, we have integrated a sentence into the minimizer digestion section detailing this fact.

Reviewer #2 Comments:
Comment:
The manuscript "SPUMONI2" by Ahmed et al presents a significant improvement over the previously published tool, SPUMONI for read classification problems including taxonomic classification, host sequence depletion, adaptive sampling of long nanopore reads, and accurate detection of contaminants in reference databases.
The authors have developed a multi-class read classifier that is both faster and more memory-efficient. By utilizing a novel sampled document array structure, the tool is capable of efficiently solving read classification problems for both short and long reads. The results show a three-fold increase in speed and a two-fold reduction in index size compared to SPUMONI. The authors also demonstrate the tool's ability to effectively identify contaminated contigs in genome assemblies, further highlighting its versatility and usefulness.
The authors have effectively validated the performance of SPUMONI2 using different datasets such as 500 Escherichia coli genomes, a mock community dataset, and the Human microbiome dataset, and compared it with SPUMONI and minimap2. They found that SPUMONI2 has a 15x faster classification speed for both long and short reads in the mock community dataset with a far smaller index size compared to minimap2.
Response:
We agree with reviewer #2’s assessment of the paper and their key takeaways.

Comment:
However, it should be noted that the improved speed and memory footprint come with a slight reduction in classification accuracy. To better evaluate the impact of minimizer digestion on classification accuracy, one suggestion would be to include additional data points for classification accuracy using additional window sizes 10, 11, and 12.

Furthermore, a deeper analysis of the regions that the tool struggles to classify correctly using SPUMONI2's minimizer digestion could provide valuable insights into the complexity of these regions.
Overall, the SPUMONI2 tool is a valuable addition to the read classification problem and the authors should be recognized for their efforts.
Response:
We agree it is important to acknowledge and measure the slight reduction in accuracy that occurs once we use minimizer digestion. We are unsure if the reviewer is referring to Figure 3; in that figure we do include classification accuracy points for w = 10, 11, and 12. They are not labeled like w = 8 and 9 but we did include a point for each window size from 8 to 24 to show the impact of the minimizer digestion of classification and we labeled the ends of the curves to help make it more understandable for what each point corresponds to. We added a sentence in Figure 3 to clarify which large window sizes were tested.
In terms of the second point, we did not conduct a specific analysis of what biologically meaningful regions might be favored or disfavored by minimizer digestion. We focused on understanding how the sparsity of the minimizer digestion affects classification, as shown in Figure 3. We expect that the randomness of the hash function ensures that there is no major systematic bias toward or against regions based on any biological label.
Further, if there were such a bias, we expect it could be addressed by simply repeating the analysis with a new selection of a hash function. That said, it is extremely common for tools using minimizer digests or indexes to use a single hash function, e.g. minimap2 and mDBG.
We included two new paragraphs in the Discussion section making this point and adding a second related point regarding how our minimizer compression scheme deals with tandem repeats.

Comment:
Move Table 2 after section 2.4.
In section 2.5, line 36, delete one "using" from a sentence.
Move Figure 4 after section 2.5
Response:
Thanks to the reviewer for the suggested revisions, we have made all those changes.
